# Supplementary material for: A robust evaluation of TDP-43, poly GP, cellular pathology and behavior in a AAV-C9ORF72 (G4C2)66 mouse model
Source: bioRxiv. 2024 Aug 27:2024.08.27.607409. Preprint. [Version 1] doi: 10.1101/2024.08.27.607409 (PMC11383318; doi:10.1101/2024.08.27.607409)
Supplement: 1 [file NIHPP2024.08.27.607409V1-supplement-1.pdf]

bioRxiv preprint doi: https://doi.org/10.1101/2024.08.27.607409; this version posted August 27, 2024. The copyright holder for this preprint (which was not certified by peer review) is the author/funder, who has granted bioRxiv a license to display the preprint in perpetuity. It is made available under aCC-BY 4.0 International license.

Supplemental Figures

bioRxiv preprint doi: https://doi.org/10.1101/2024.08.27.607409; this version posted August 27, 2024. The copyright holder for this preprint (which was not certified by peer review) is the author/funder, who has granted bioRxiv a license to display the preprint in perpetuity. It is made available under aCC-BY 4.0 International license.

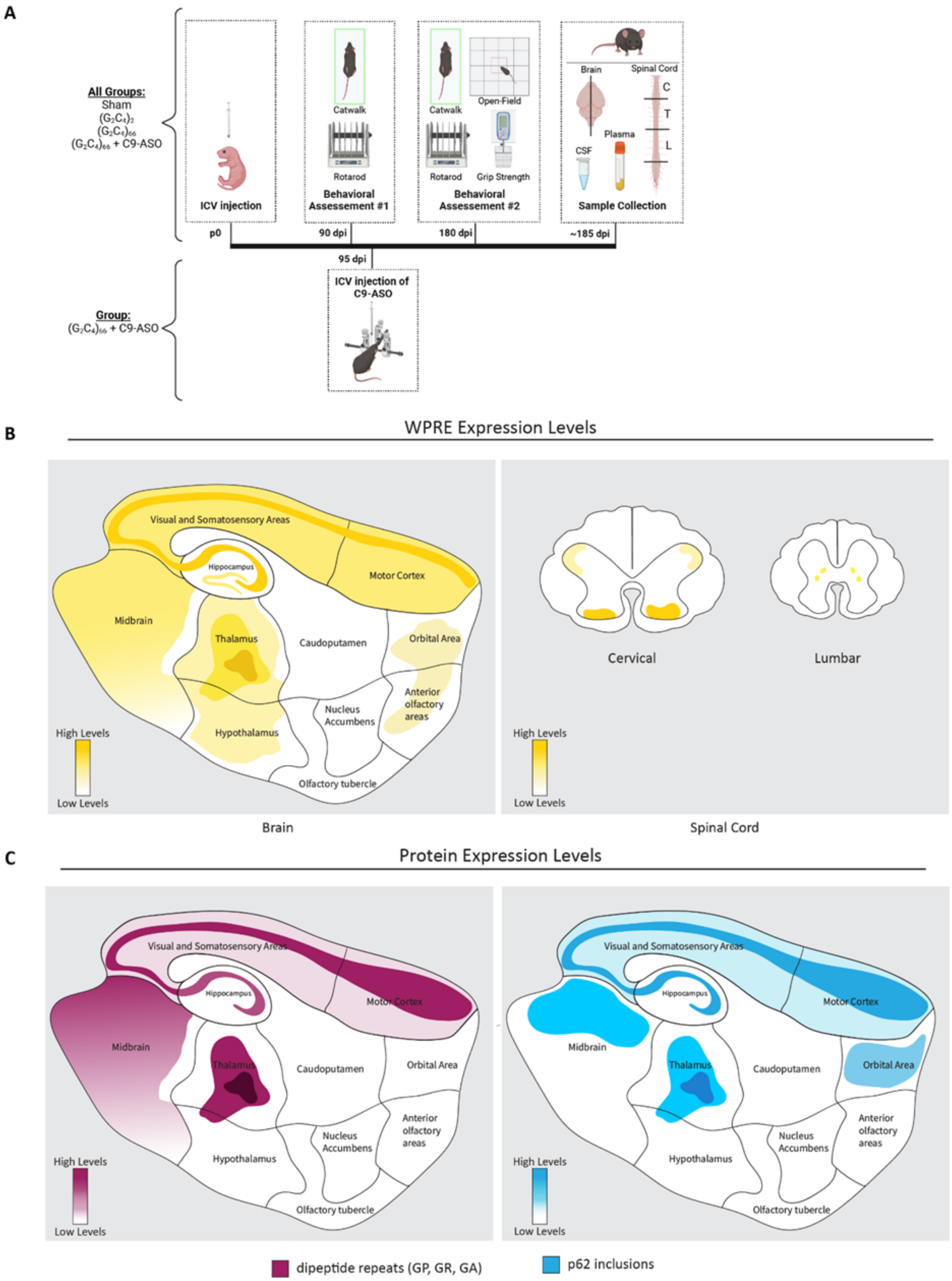

Supplemental Figure 1. (A) Schematic overview of the study design. Cartoon representation of spatial viral RNA expression **(B)** and protein expression levels of dipeptide repeat expression

bioRxiv preprint doi: https://doi.org/10.1101/2024.08.27.607409; this version posted August 27, 2024. The copyright holder for this preprint (which was not certified by peer review) is the author/funder, who has granted bioRxiv a license to display the preprint in perpetuity. It is made available under aCC-BY 4.0 International license.

(purple) and p62 inclusions (blue) in **(C)**. Both DPR expression and p62 inclusions are localized to the areas where viral $(G_4C_2)_{66}$ RNA is expressed.

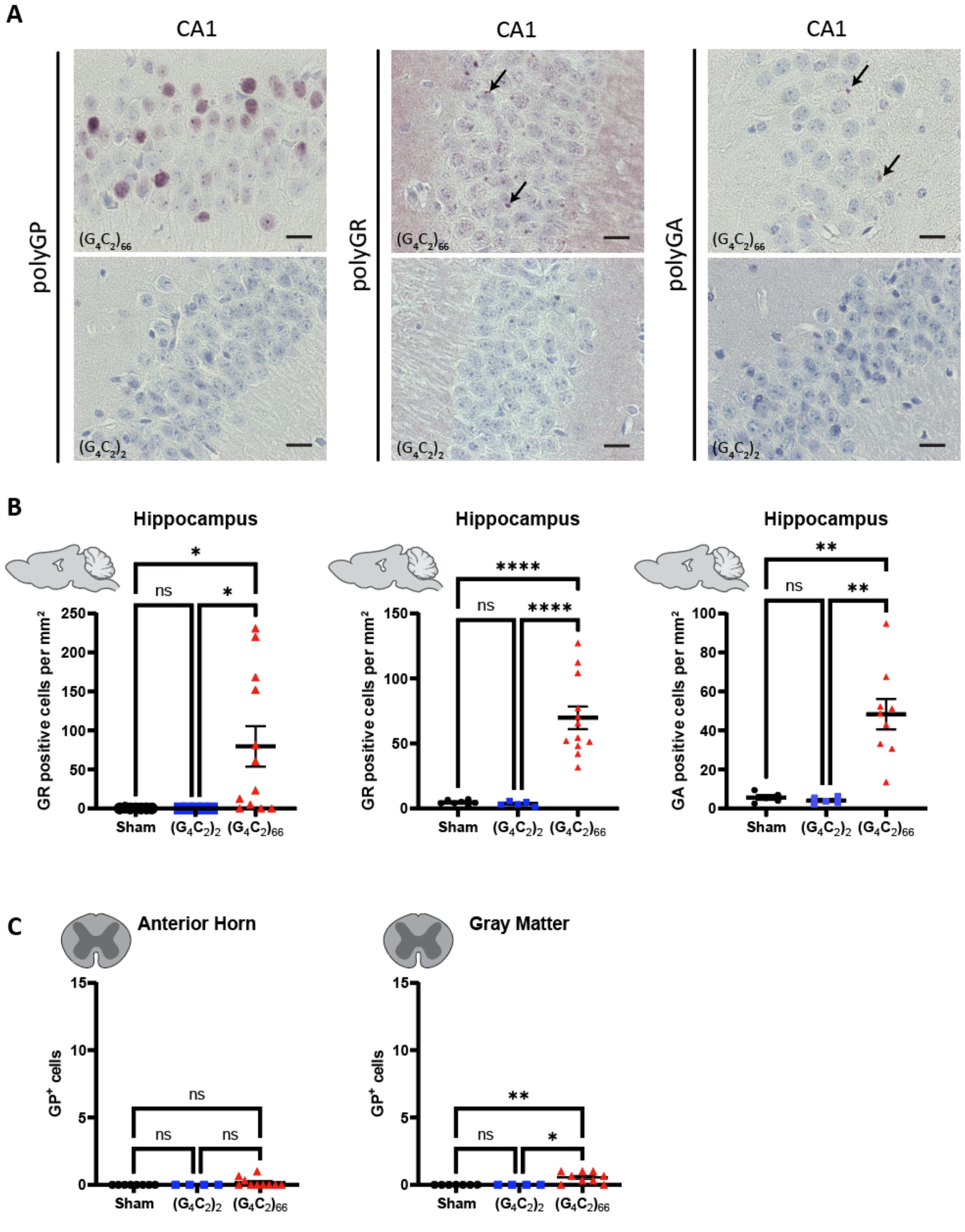

bioRxiv preprint doi: https://doi.org/10.1101/2024.08.27.607409; this version posted August 27, 2024. The copyright holder for this preprint (which was not certified by peer review) is the author/funder, who has granted bioRxiv a license to display the preprint in perpetuity. It is made available under aCC-BY 4.0 International license.

Supplemental Figure 2. $(G_4C_2)_{66}$ mice have DPR expression in the hippocampus. (A) Representative images of CA1 region from brain sections stained against polyGP, polyGR, and polyGA. Scale bars 20 µm. **(B)** Quantification of sections stained against polyGP, polyGR, and polyGA (sham n=14, $(G_4C_2)_2$ n=12, $(G_4C_2)_{66}$ n=12). One-way Welch ANOVA analysis of polyGP, polyGR, and polyGA positive cell number was performed (p=0.0021 for polyGP, p<0.0001 for polyGR, p=0.0001 for polyGA) followed by Tukey's multiple comparison test. polyGP: sham vs $(G_4C_2)_2$ p=0.9993, sham vs $(G_4C_2)_{66}$ p=0.0303 and $(G_4C_2)_2$ vs $(G_4C_2)_{66}$ p=0.0304, polyGR: sham vs $(G_4C_2)_2$ p=0.6542, sham vs $(G_4C_2)_{66}$ p<0.0001 and $(G_4C_2)_2$ vs $(G_4C_2)_{66}$ p<0.0001, polyGA: sham vs $(G_4C_2)_2$ p=0.6636, sham vs $(G_4C_2)_{66}$ p=0.0018 and $(G_4C_2)_2$ vs $(G_4C_2)_{66}$ p=0.0013, error bars = SEM. **(C)** Quantification of lumbar spinal cord sections stained against polyGP (sham n=7, $(G_4C_2)_2$ n=4, $(G_4C_2)_{66}$ n=9). A Kruskal-Wallis test of the lumbar spinal cord gray matter (p=0.0015) and anterior horn (p=0.1870) dataset was performed, followed by Dunn's multiple comparisons test (gray matter: sham vs. $(G_4C_2)_2$ p>0.9999, sham vs. $(G_4C_2)_{66}$ p=0.0064, $(G_4C_2)_2$ vs. $(G_4C_2)_{66}$ p=0.0301; anterior horn: sham vs. $(G_4C_2)_2$ p>0.9999, sham vs. $(G_4C_2)_{66}$ p=0.2180, $(G_4C_2)_2$ vs. $(G_4C_2)_{66}$ p=0.4504) , error bars = SEM.

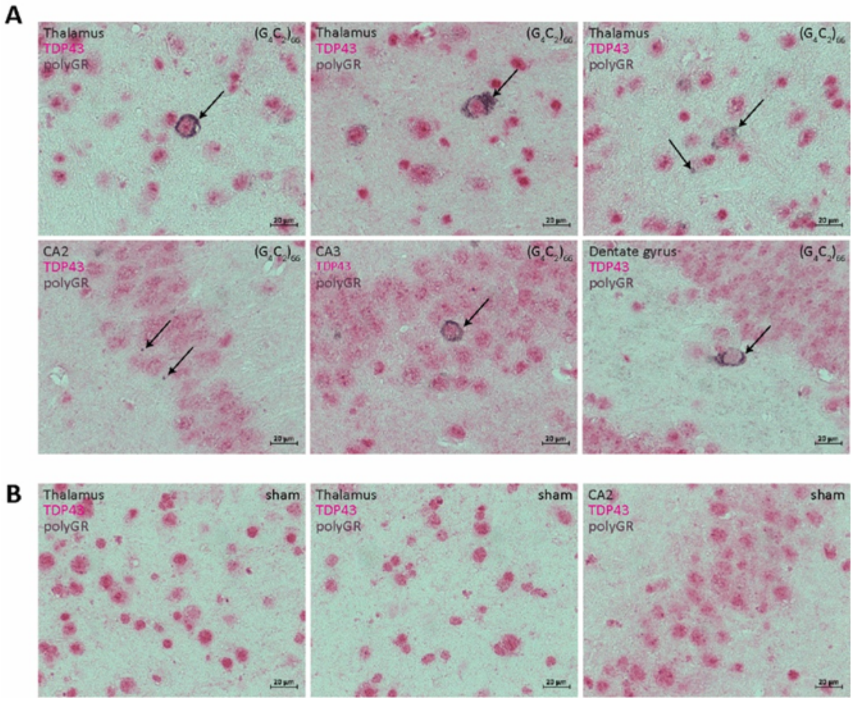

Supplemental Figure 3. TDP-43 does not mislocalize in polyGR positive cells. (A) Representative images of Thalamus, CA1, CA3, and Dentate Gyrus brain regions in $(G_4C_2)_{66}$ mice co-labled with TDP-43 (pink) and polyGR (brown). Scale bars 20 µm. No mislocalization of TDP-43 into the cytoplasm is observed in cells positive for TDP-43 and polyGR (denoted by black arrows). **(B)** Representative images of Thalamus and CA2 brain regions in sham mice co-labled with TDP-43 (pink) and polyGR (brown). No polyGR positive cells are present in sham tissue. Scale bars 20 µm.

bioRxiv preprint doi: https://doi.org/10.1101/2024.08.27.607409; this version posted August 27, 2024. The copyright holder for this preprint (which was not certified by peer review) is the author/funder, who has granted bioRxiv a license to display the preprint in perpetuity. It is made available under aCC-BY 4.0 International license.

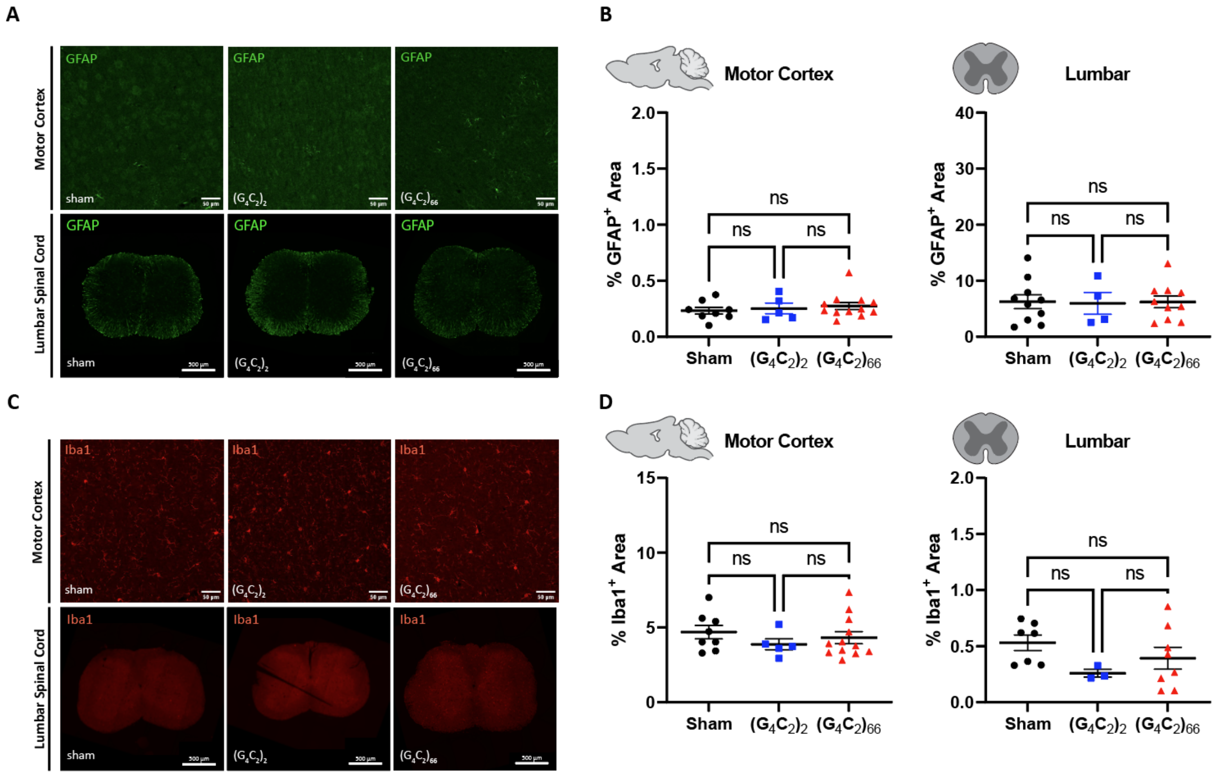

Supplemental Figure 4. Analysis of gliosis in the $(G_4C_2)_{66}$ mouse model by immunohistochemistry. (A) Representative images of immunohistochemistry analysis of GFAP expression within the motor cortex and lumbar spinal cord. Motor cortex scale bars 50 μm and lumbar spinal cord scale bars 200 μm. **(B)** Quantification of % GFAP positive area within cortex and cervical spinal cord (sham n=8 for motor cortex and 10 for spinal cord, $(G_4C_2)_2$ n=5 for, motor cortex and 4 for spinal cord, $(G_4C_2)_{66}$ n=12 for motor cortex and 10 for spinal cord). One-way ANOVA analysis of the motor cortex dataset was performed (p=0.6690), followed by Tukey's multiple comparisons test: sham vs. $(G_4C_2)_2$ p=0.9433, sham vs. $(G_4C_2)_{66}$ p=0.6479, $(G_4C_2)_2$ vs. $(G_4C_2)_{66}$ p=0.9077. One-way ANOVA analysis of the lumbar spinal cord dataset was performed (p=0.9891), followed by Tukey's multiple comparisons test: sham vs. $(G_4C_2)_2$ p=0.9888, sham vs. $(G_4C_2)_{66}$ p=0.9998, $(G_4C_2)_2$ vs. $(G_4C_2)_{66}$ p=0.9909, error bars = SEM. **(C)** Representative images of immunohistochemistry analysis of Iba1 expression within motor cortex and lumbar spinal cord. Cortex scale bars 50 μm and lumbar spinal cord scale bars 200 μm. **(D)** Quantification of % Iba1 positive area within motor cortex and lumbar spinal cord (sham n=8 for motor cortex and 7 for spinal cord, $(G_4C_2)_2$ n=5 for motor cortex and 3 for spinal cord, $(G_4C_2)_{66}$ n=12 for motor cortex and 8 for spinal cord). One-way ANOVA analysis of the motor cortex dataset was performed (p=0.5259), followed by Tukey's multiple comparisons test: sham vs. $(G_4C_2)_2$ p=0.4978, sham vs. $(G_4C_2)_{66}$ p=0.7929, $(G_4C_2)_2$ vs. $(G_4C_2)_{66}$ p=0.7852. One-way ANOVA analysis of the lumbar spinal cord dataset was performed (p=0.2099), followed by Tukey's multiple comparisons test: sham vs. $(G_4C_2)_2$ p=0.2085, sham vs. $(G_4C_2)_{66}$ p=0.4674, $(G_4C_2)_2$ vs. $(G_4C_2)_{66}$ p=0.6513, error bars = SEM.

bioRxiv preprint doi: https://doi.org/10.1101/2024.08.27.607409; this version posted August 27, 2024. The copyright holder for this preprint (which was not certified by peer review) is the author/funder, who has granted bioRxiv a license to display the preprint in perpetuity. It is made available under aCC-BY 4.0 International license.

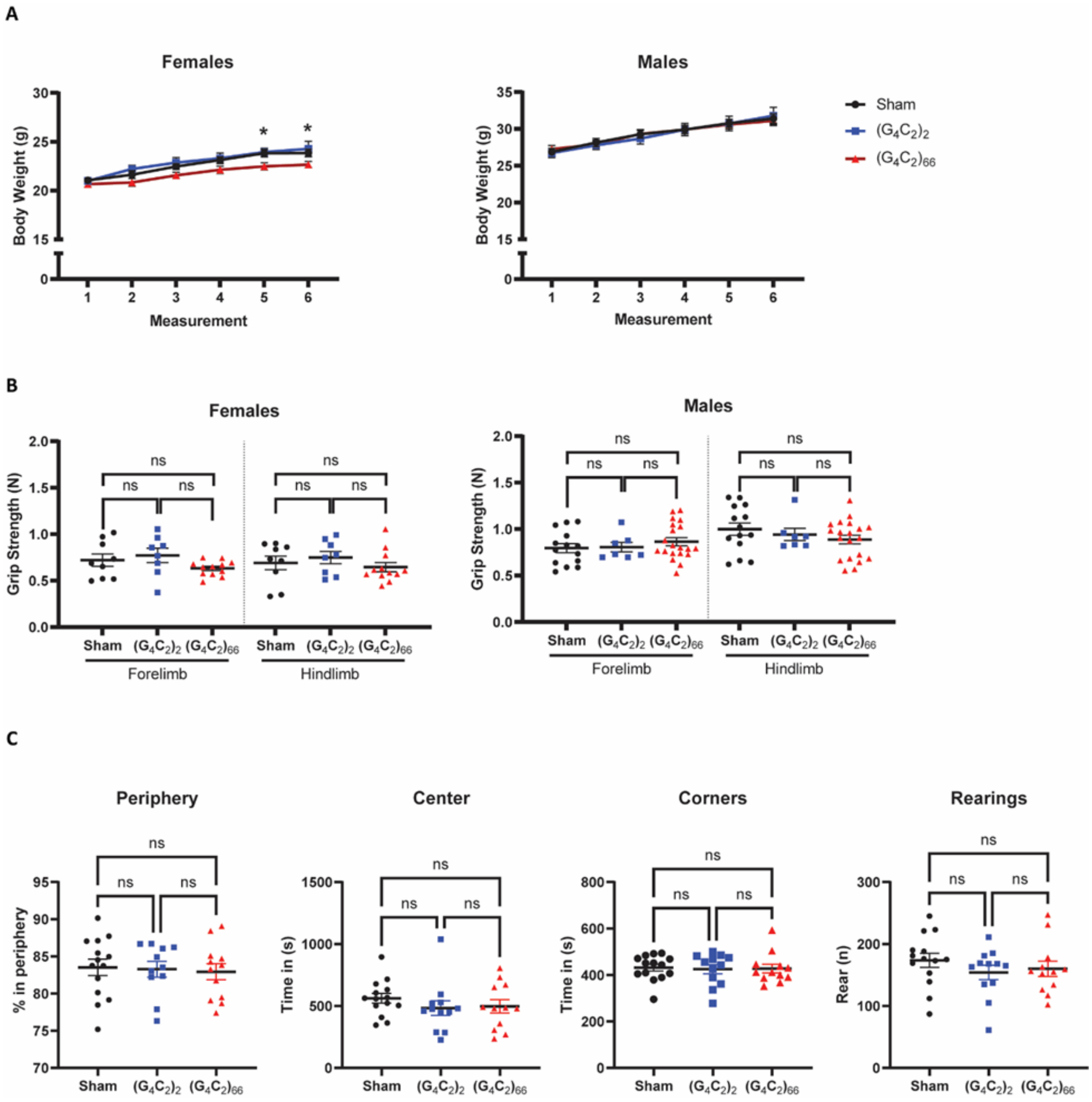

Supplemental Figure 5. $(G_4C_2)_{66}$ mice do not have behavioral deficits. (A) Bodyweight tracking of mice (females: sham n=18, $(G_4C_2)_2$ n=8, $(G_4C_2)_{66}$ n=15, males: sham n=14, $(G_4C_2)_2$ n=10, $(G_4C_2)_{66}$ n=20). A two-way repeated-measures ANOVA analysis of the female dataset was performed (p=0.0573), followed by Dunnett's multiple comparisons test where a significant difference was observed at the fifth (sham vs. $(G_4C_2)_{66}$ p=0.0276) and sixth sham vs. $(G_4C_2)_{66}$ p=0.0479 measurement. A two-way repeated-measures ANOVA analysis of the male dataset was performed (p=0.9917), followed by Dunnett's multiple comparisons test but no significant difference was found between the groups for any of the bodyweight measurements, error bars = SEM. **(B)** Grip strength behavioral analysis at 90 dpi in female (sham n=9, $(G_4C_2)_2$ n=8, $(G_4C_2)_{66}$ n=12) and male mice (sham n=14, $(G_4C_2)_2$ n=7, $(G_4C_2)_{66}$ n=20). One-way ANOVA analysis of the female dataset was performed (forelimb: p=0.1871, hindlimb: p=0.5037), followed by Tukey's multiple comparisons test: sham vs. $(G_4C_2)_2$ (forelimb: p=0.8087, hindlimb: p=0.8097), sham vs.

bioRxiv preprint doi: https://doi.org/10.1101/2024.08.27.607409; this version posted August 27, 2024. The copyright holder for this preprint (which was not certified by peer review) is the author/funder, who has granted bioRxiv a license to display the preprint in perpetuity. It is made available under aCC-BY 4.0 International license.

$(G_4C_2)_{66}$ (forelimb: p=0.4616, hindlimb: p=0.8514), $(G_4C_2)_2$ vs. $(G_4C_2)_{66}$ (forelimb: p=0.1808, hindlimb: p=0.4722). One-way ANOVA analysis of the male dataset was performed (forelimb: p=0.5226, hindlimb: p=0.3495), followed by Tukey's multiple comparisons test: sham vs. $(G_4C_2)_2$ (forelimb: p=0.9918, hindlimb: p=0.8354), sham vs. $(G_4C_2)_{66}$ (forelimb: p=0.5329, hindlimb: p=0.3175), $(G_4C_2)_2$ vs. $(G_4C_2)_{66}$ (forelimb: p=0.7492, hindlimb: p=0.8414) , error bars = SEM. **(C)** Time spent in periphery, center, corners and number of rearing analyses in open-field test at 180 dpi (n=14 sham, n=12 $(G_4C_2)_2$, n=12 $(G_4C_2)_{66}$). One-way ANOVA analyses of time spent in periphery, in center, in corners, and the number of rearing were performed (p=0.9207 for time spent in periphery, p=0.4894 for time spent in center, p=0.9647 for time spent in corners, and p=0.4693 for number of rearings) followed by Tukey's multiple comparison test. Periphery: sham vs $(G_4C_2)_2$ p=0.9840, sham vs $(G_4C_2)_{66}$ p=0.9130 and $(G_4C_2)_2$ vs $(G_4C_2)_{66}$ p=0.9741, Center: sham vs $(G_4C_2)_2$ p=0.5099, sham vs $(G_4C_2)_{66}$ p=0.6300 and $(G_4C_2)_2$ vs $(G_4C_2)_{66}$ p=0.9807, Corners: sham vs $(G_4C_2)_2$ p=0.9620, sham vs $(G_4C_2)_{66}$ p=0.9864 and $(G_4C_2)_2$ vs $(G_4C_2)_{66}$ p=0.9941, Rearings: sham vs $(G_4C_2)_2$ p=0.4598, sham vs $(G_4C_2)_{66}$ p=0.6833 and $(G_4C_2)_2$ vs $(G_4C_2)_{66}$ p=0.9339, error bars = SEM.
